# A Dual-Pronged Hafnia–Prodrug–Lipid Nanoplatform Coupling Radiosensitization and DNA Homologous Recombination Inhibition

**DOI:** 10.1021/acsnanomed.6c00022

**Published:** 2026-04-14

**Authors:** David Skrodzki, Pranay Saha, Matthew Molinaro, Nada Maher, Shraddha Krishnakumar, Nivetha Gunaseelan, Mecit Altan Alioglu, Oguzhan Colak, Gabriela Cepeda, Parikshit Moitra, Timothy M. Fan, Dipanjan Pan

**Affiliations:** † Department of Materials Science and Engineering, The Pennsylvania State University, University Park, Pennsylvania 16802, United States; ‡ Department of Nuclear Engineering, 311285 The Pennsylvania State University, University Park, Pennsylvania 16802, United States; § Department of Engineering Science and Mechanics, The Pennsylvania State University, University Park, Pennsylvania 16802, United States; ∥ Department of Biomedical Engineering, The Pennsylvania State University, University Park, Pennsylvania 16802, United States; ⊥ Huck Institutes of Life Sciences, 101 Huck Life Sciences Building, University Park, Pennsylvania 16802, United States; # Department of Chemistry, The Pennsylvania State University, University Park, Pennsylvania 16802, United States; ∇ Department of Veterinary Clinical Medicine, University of Illinois Urbana−Champaign, Urbana, Illinois 61802, United States; ○ Cancer Center at Illinois, University of Illinois Urbana−Champaign, Urbana, Illinois 61802, United States

**Keywords:** Combinatorial nanoscale strategies, Cancer, Radiotherapy, Chemotherapy, High-Z radiosensitization, Prodrugs

## Abstract

Emerging nanomedicine strategies have established clinical translation and the ability to offset conventional therapeutic challenges. This study aimed to report the development and analysis of a nanoscale material that functioned as both a high-Z radiosensitizer and a prodrug delivery system. In this study, a nanoscale platform using two complementary radiosensitizing mechanisms was designed and developed to overcome nonspecific toxicity, which is a challenge in both chemotherapy and radiotherapy. High-Z physical radiosensitization was coupled with the inhibition of a critical DNA double-strand break repair mechanism, homologous recombination, through a lipid nanoparticle decorated with ultrasmall Hafnia nanoparticles and loaded with an amphiphilic prodrug. In vitro and in vivo studies demonstrated significant enhancement of therapeutic effects, cell killing, or tumor regression, respectively, versus radiation alone. These results support the use of this combination system as a practical strategy for maintaining treatment efficacy at modest doses of radiation and offset associated adverse effects.

## Graphical abstract

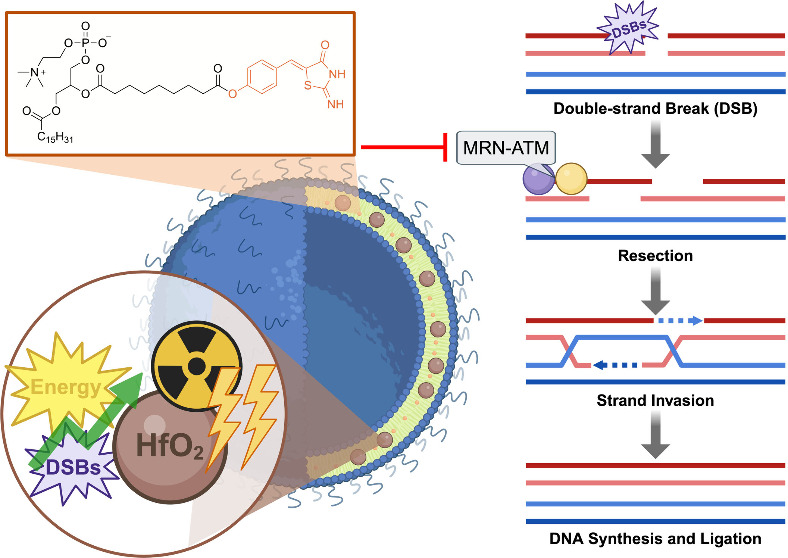

## Introduction

Combinatorial nanoscale strategies transform cancer therapy by integrating targeted drug delivery, radiosensitization, and spatiotemporally controlled release into a single therapeutic platform. Despite global advances in cancer diagnosis and treatment, >20 million new cases are projected annually by 2025, underscoring the need for safer and more effective interventions.^1^ Conventional radiotherapy (RT) and chemotherapy remain standard treatments but are limited by systemic toxicity and poor tumor selectivity.^2^ RT is extensively used in both neoadjuvant and adjuvant settings, and more than half of all patients with cancer receive RT during the course of their care regimen.^3–5^ Cell killing and tumor shrinkage are achieved through the deposition of energy in the cell environment, resulting in damage to critical cellular components, such as DNA. Although effective against malignant cells, ionizing radiation unavoidably impacts adjacent healthy tissues, resulting in off-target damage and clinically significant side effects, including inflammation, fatigue, nausea, and tissue-specific toxicity.^6^ Through high-Z radiosensitization, nanomedicine enables tumor-selective amplification of radiation effects by concentrating high-atomic-number nanoparticles within malignant tissues, thereby improving RT efficacy while limiting normal-tissue toxicity.^4,7^ Therefore, more modest radiation doses can be used without compromising the curative efficacy of RT. Similarly, chemotherapy has poor selectivity, owing to systemic delivery and the inherently cytotoxic nature of its agents. Nanoscale drug delivery vehicles have demonstrated the capacity to offset the nonspecific toxicity and risk of adverse effects by increasing drug delivery and payload to the target site, thereby concentrating drugs in tumors and minimizing off-target exposure.^8–11^ Prodrugs, pharmacologically inert molecules that require metabolic or chemically triggered conversion to generate active therapeutics, offer an additional strategy to enhance the therapeutic index of chemotherapy.^12–16^ The complementary integration of prodrug design with nanomedicine suggests that their concurrent implementation may further improve the safety, selectivity, and efficacy of chemotherapeutic agents.

Despite a strong mechanistic rationale for combining homologous recombination (HR) inhibition with RT, its clinical translation is limited by fundamental drug delivery constraints. Many HR inhibitors exhibit poor stability and rapidly dissociate from nanoparticle carriers during intravascular circulation, thereby undermining tumor exposure and therapeutic synergy with ionizing radiation. To address these limitations, our group has previously established lipase-labile phospholipid prodrugs as a strategy to stabilize therapeutic agents within nanocarriers while enabling enzyme-triggered release into the tumor microenvironment.^10,16–18^ This method combines conditional prodrug activation with nanoscale delivery to directly tackle the intrinsic lack of tumor selectivity in systemic chemotherapy, establishing a platform for coordinated spatial and temporal HR reduction under radiation exposure.

Herein, we aimed to report the development and analysis of a nanoscale material that functioned as both a high-Z radiosensitizer and a prodrug delivery system (Figure 1). These two capabilities possessed complementary mechanisms, namely, enhanced induction of DNA double-strand breaks (DSBs) and suppression of the principal DSB repair pathway. The nanomaterial design consisted of a lipid nanoparticle (LNP) incorporating hafnium oxide nanoparticles that displayed hydrophobic characteristics and were tethered within the nonpolar portion of the lipid bilayer (HfLNP). LNPs offered a biomimetic interface that enhanced the compatibility with biological systems and aided in the loading of the amphiphilic prodrug.^19^ The prodrug proMirin (pM) (*Z*)-2-((9-(3-((2-imino-4-oxothiazolidin-5-ylidene)­methyl)­phenoxy)-9-oxononanoyl)­oxy)-3-(palmitoyloxy)­propyl­(2-(trimethylammonio)­ethyl) phosphate is a covalently modified version of mirin [Z-5-(4-hydroxybenzylidene)-2-imino-1,3-thiazolidin-4-one], an established DNA damage repair inhibitor small molecule drug. Direct radiation-induced damage, the most lethal pathway, is based on energy deposition in DNA and the resulting formation of DNA–DNA and DNA–protein cross-links, single-strand breaks, and DSBs.^20^ DSBs are considered to be the predominant contribution to loss in cell viability and lethality.^20–22^ DSB repair proceeds through two major processes: nonhomologous end joining and homologous recombination (HR).^23^ HR occurs during the S and G2 phase of the cell cycle and is employed in response to DSBs directly induced by irradiation (IR) or those consequent of replication fork stalling.^24^ Mirin interferes with the Mre11-Rad50-Nbs1 (MRN)-ataxia-telangiectasia mutated (ATM) pathway, a critical process for DNA damage recognition, regulation of cell cycle checkpoints, and repair mechanism signaling.^25,26^ The MRN complex recognizes DSBs and proceeds to signal and activate ATM. In turn, ATM phosphorylates critical repair and cell cycle arrest proteins. Mirin inhibits DSB repair by interfering with the MRN-dependent ATM activation. This outcome is attributed to the inhibition of Mre11 exonuclease activity, G2/M checkpoint, and thus homologous-dependent repair.^27^ The prodrug was designed to be released from the bilayer within the tumor microenvironment via enzymatic cleavage by phospholipase A2 at the ester position SN-2 position ester bond. The ester bond connecting the active drug molecule to the SN-2 fatty acid residue of the phospholipid served as a cleavage site for acidic hydrolysis in the lower pH of the tumor microenvironment, thereby activating the pharmacological agent. Dual-pronged prodrug-based chemotherapeutic and high-Z radiosensitization effects were studied using in vitro and in vivo models, revealing substantial therapeutic effects.

**Figure 1:**
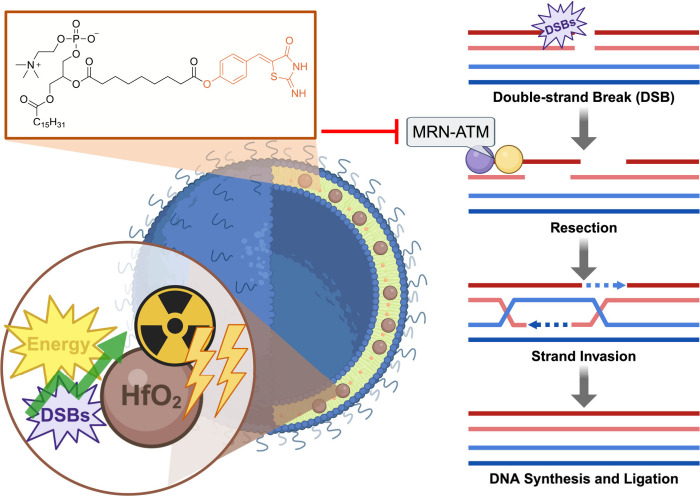
Schematic representation of dual-pronged radiosensitization strategy and depiction of a prodrug-bearing lipid nanoparticle decorated with Hafnia nanoparticles.

## Results

### NP Synthesis and Characterization

Hafnium oxide nanoparticles 3–5 nm in diameter were synthesized using a routine precipitation method and characterized using standard characterization techniques. The size and crystallinity were confirmed by transmission electron microscopy (TEM) (Figure 2A). X-ray diffraction verified the crystalline nature, and the diffraction pattern matched that of a known monoclinic structure (PDF 04–072–0468) (Figure 2B). The elemental composition was assessed by using high-angle annular dark-field scanning TEM and energy-dispersive spectroscopy (Figure 2C, D). The stability of the particles in water was studied by using zeta potential measurements (Figure S1). The particles exhibited poor dispersion and relative instability with a ξ = −7.6 ± 17.3. This poor colloidal stability for bare hafnium oxide nanoparticles matches previous reports.^28^ At a near neutral pH, the particles exhibit a surface charge near zero, as verified experimentally. Furthermore, hafnia is known to have a relatively high Hamaker constant and thus stronger van der Waals attraction between particle bodies.^29^ Based on the Derjagiun-Landau-Verwey-Overbeek (DLVO) theory, the poor dispersion observed is attributed to the lack of strong interparticle electrostatic repulsion coupled with higher van der Waals attractive forces.

**Figure 2:**
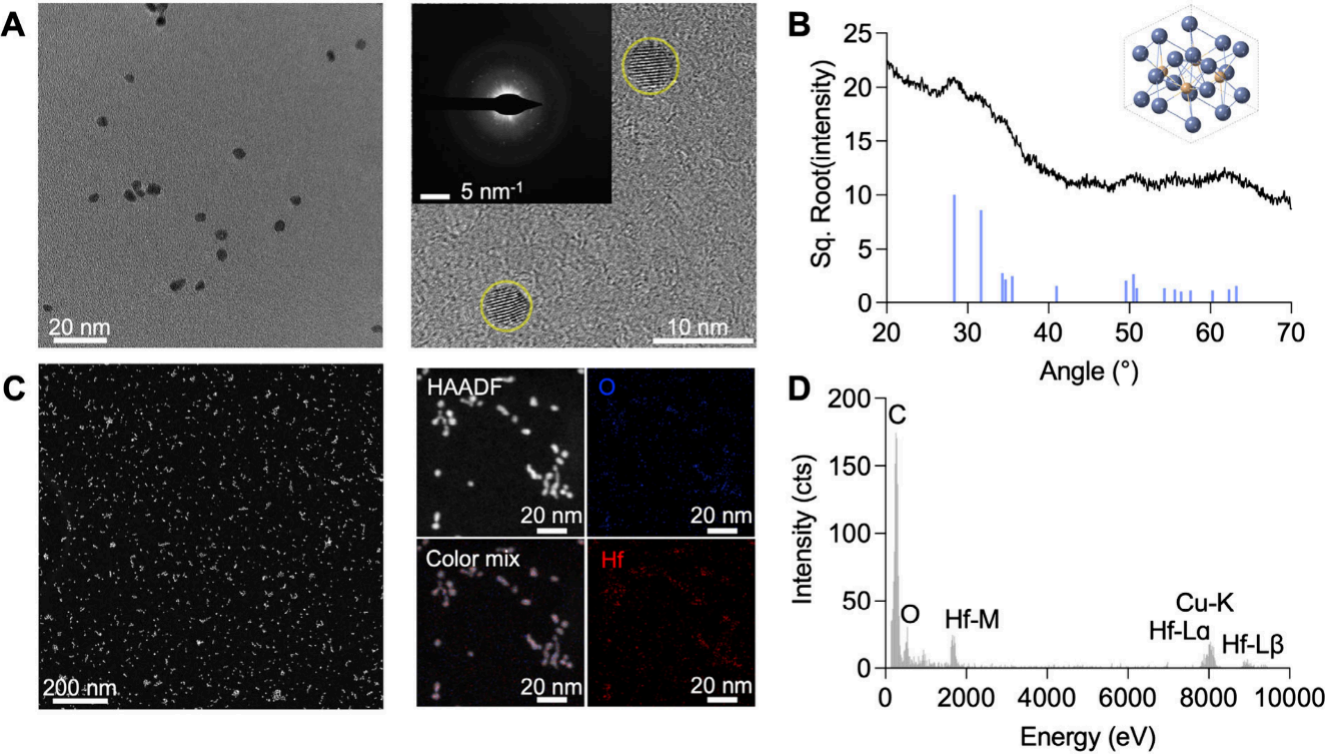
Size and crystallographic characterization of nanoparticles (NPs). (A) Transmission electron microscopy (TEM) of dispersed NPs (left) and high-resolution image depicting lattice fringes and an inlaid selected area electron diffraction pattern (right). (B) X-ray diffraction pattern of particles with an inlaid modeled structure. (C) High-angle annular dark-field scanning TEM image showing lack of aggregation (left) and higher magnification image with elemental mapping (right). (D) Energy-dispersive X-ray spectroscopy spectra acquired for elemental mapping in (C).

X-ray photoelectron spectroscopy confirmed the composition of the hafnium oxide (Figure 3A). The hydrodynamic size was ascertained by dynamic light scattering and provided results consistent with those of anhydrous methods such as TEM (Figure 3B). The LNP, decorated with NPs, exhibited an increase in hydrodynamic size and a peak at approximately 20 nm (Figure 3C). The hydrodynamic size as a function of intensity is provided in Figure S2. The mean zeta potential is −25 mV, indicating colloidal stability in terms of DLVO theory (Figure 3D). Osmium tetroxide staining was used to visualize the presence and structure of the self-assembled organic carriers of the NPs and the prodrug under TEM. A representative image is provided in Figure S3. The thin film hydration coupled with multiple freeze–thaw, sonicate–vortex cycles yielded relatively uniform HfLNPs (Figure 3E).

**Figure 3:**
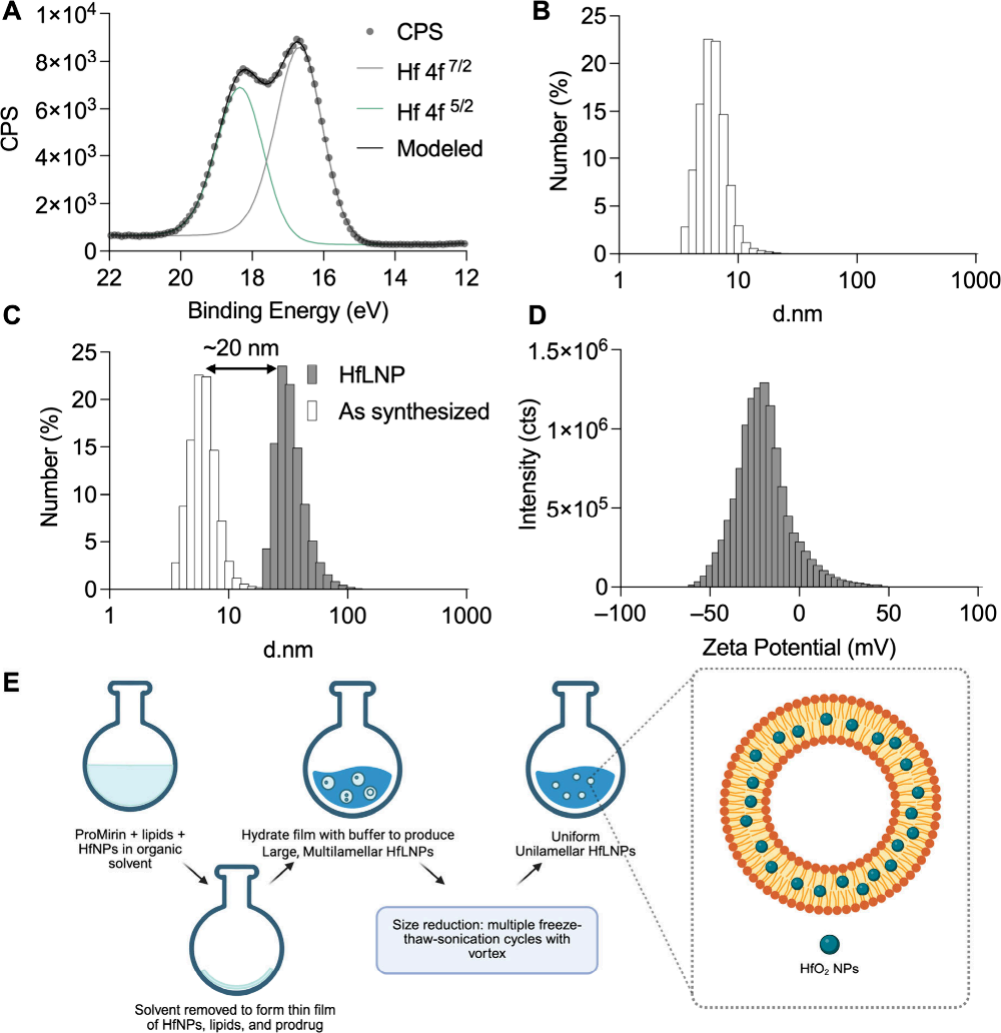
(A) X-ray photoelectron spectroscopy analysis of as-synthesized Hafnia particles. (B) Dynamic light scattering (DLS) of as-synthesized Hafnia particles (polydispersity index [PDI] = 0.198). (C) DLS of HfLNPs (PDI = 0.215) as compared with as synthesized. (D) Zeta potential of phospholipid-embedded particles. (E) Schematic representation of ProMirin-loaded HfLNP generation.

### Prodrug Synthesis, Loading, and Release

Prodrug synthesis was validated through electrospray ionization-mass spectrometry (ESI-MS) and proton nuclear magnetic resonance (Figures S4, S5). Prodrug loading into the amphiphilic LNPs was quantified using ultrahigh performance liquid chromatography coupled with tandem mass spectrometry. The analysis found 103.7 ug of prodrug per 2 mg of Hf particles (0.052 ug PM/ug HfNP) or a loading efficiency of approximately 30% (Figure S6). Prodrug release within a simulated tumor microenvironment was evaluated through an experiment detailed in detail and graphically represented in Figure S7. In brief, the prodrug at equivalent concentration was incubated with esterase at a pH of 6.8, whereas the control group lacked esterase and was maintained at pH 7.4. Both groups were incubated for 24 h under dialysis, and the dialysate was examined by ESI-MS. The active form of mirin was observed only in the samples containing the enzyme, supporting the specific release of the prodrug under intended conditions.

### In Vitro Studies

The biocompatibility and radio-enhancing effects of the NP system were evaluated using cell viability and proliferation assays. The NPs without the prodrug exhibited excellent biocompatibility, with cell viability remaining >85% for the tested concentration range until they were activated into a cytotoxic enhancer through IR (Figure 4A). In contrast, the cytotoxicity of the prodrug was demonstrated with and without radiation (Figure 4B), supporting the biological activity of DNA repair inhibition as the genetic instability inherent to cancer cells experienced a lack of repair. Clonogenic assays were used to assess the long-term radiation-induced effects (Figure 4C). These results corresponded with those of the cell viability studies, demonstrating that the prodrug displayed significant cytotoxic properties and that the particles facilitated radioenhancement.

**Figure 4:**
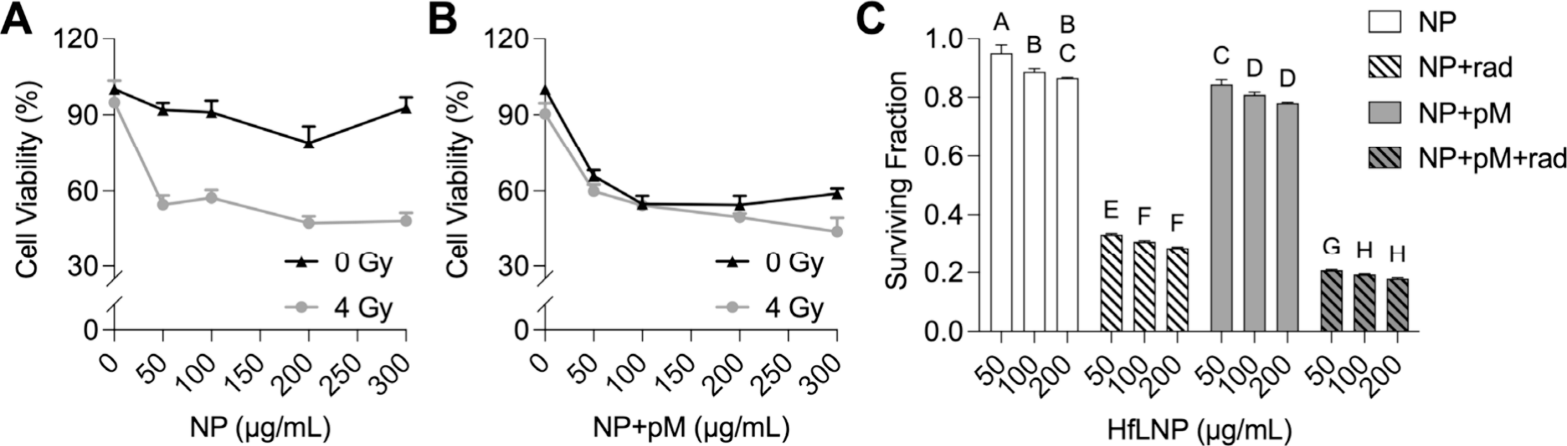
Dose–response in vitro studies with HCT116 cell line. (A) 3-(4,5-Dimethylthiazol-2-yl)-2,5-diphenyltetrazolium bromide (MTT) assay of a nanoparticle (NP) without the prodrug. (B) MTT assay of an NP with the prodrug. (*n* = 4). (C) Clonogenic assay results highlighting the effect of prodrug inclusion with a radiation dose of 4 Gy and without (*n* = 4 ± 2). Error bars plotted as the standard error of the mean for (A) and (B).

Radiosensitization mechanistic studies include the γ-H2AX flow cytometry assay to evaluate DSB formation and reactive oxygen species (ROS) generation enhancement via the 2′,7′-dichlorodihydrofluorescein diacetate:rapid sulforhodamine B assay (Figure 5A, B). The combination system exhibited a significantly greater gamma-H2AX signal intensity and an approximately 2-fold increase in ROS generation. NP internalization kinetics and intracellular localization were evaluated using inductively coupled plasma mass spectrometry (ICP-MS) and resin-embedded TEM, respectively (Figure 5C, D). ICP-MS results of Hf concentration normalized to cells count following preset incubation times depicted no statistically significant difference across time intervals, implying cellular uptake reached saturation within 2 h (Figure 5C). Furthermore, particles were observed as both well-dispersed entities and aggregated clusters within the cells (Figure 5D). The latter observation may be attributed to the degradation of the LNP structure promoting HfNP colloidal stability, as expected within a cellular environment.^30^ On the other hand, the dispersed particle bodies are likely to be associated with LNPs not yet degraded to the point where in vitro colloidal stability is lost. The physical nature of high-Z radiosensitization benefits from dispersed particles with more surface area for ionization events.^31^ Therefore, further research aimed at optimizing the LNP formulation for improved resistance to intracellular degradation is a potential direction for subsequent investigations. The crystallinity and structural stability of the particles were clearly observed by using high-resolution TEM.

**Figure 5:**
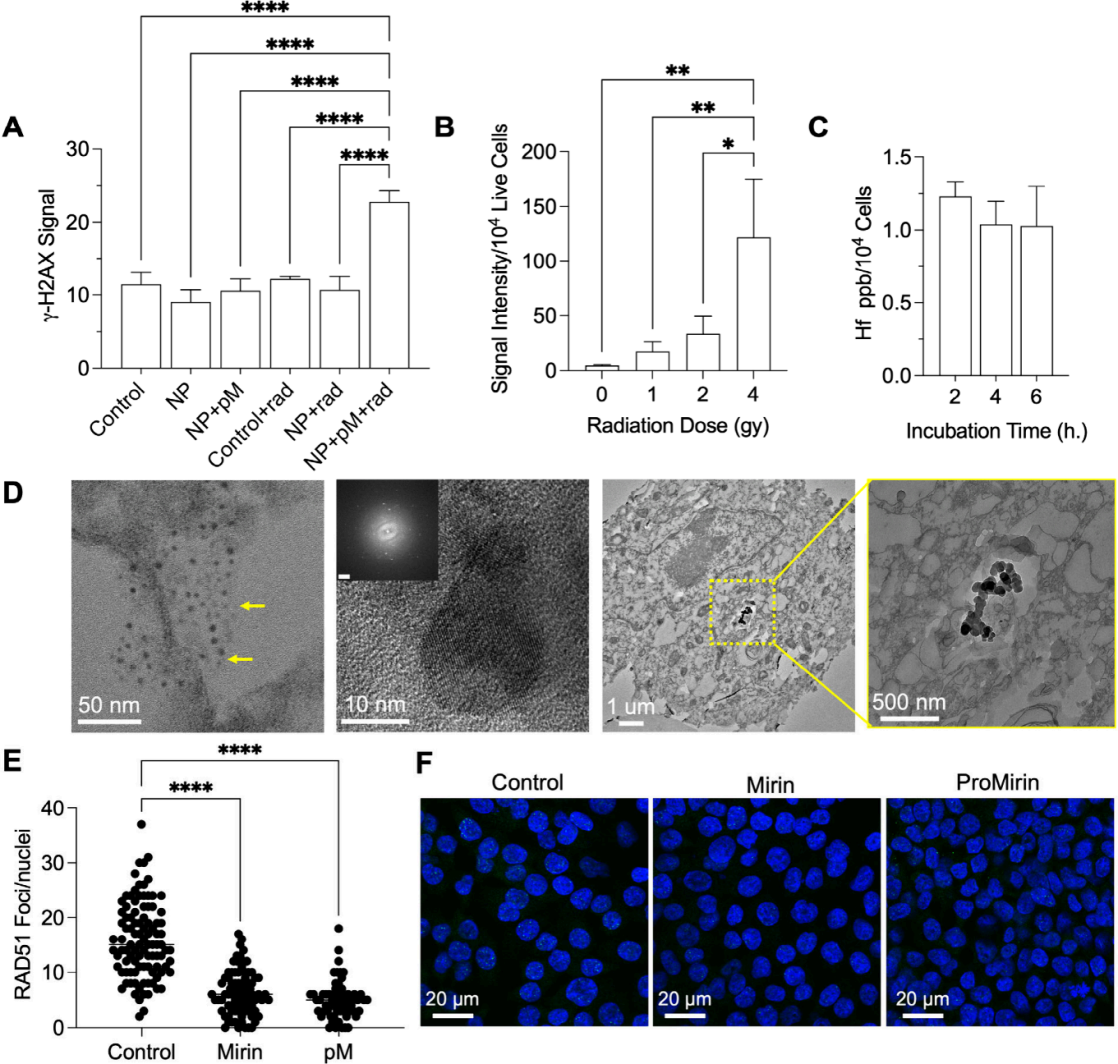
Mechanistic in vitro studies in the HCT116 cell line. (A) γ-H2AX assay evaluating double-strand breaks using flow cytometry. Significance denoted in comparison to NP+pM+rad (*n* = 3). (B) Relative reactive oxygen species (ROS) generation. Results depicted normalized to no radiation groups ROS signal intensity (*n* = 3). (C) Kinetic cellular uptake study (*n* = 4 ± 2). (D) Resin-embedded HCT116 cells treated NP+pM depicting Hafnia nanoparticles within cells. RAD51 immunofluorescence evaluating the prodrug mechanism integrity versus the original form of the drug. Samples treated with 10 uM drug/prodrug where applicable and 4 Gy radiation. (E) Comparison of foci/cell data collected from three images per sample. Counting performed using CellProfiler. (F) Representative confocal microscopy images. Nuclei are colored blue and RAD51 foci green.

The integrity of the biological mechanism of the prodrug was evaluated using a RAD51 assay (Figure 5E, F), and the presence of RAD51 foci (green) within the nuclei (blue) suggests successful initiation of the MRN-ATM pathway critical for HR. Notably, the effectiveness of the prodrug and drug was not found to be statistically different.

### In Vivo Tumor Xenograft Model Study

The promising in vitro results prompted us to evaluate the nanosystem in vivo. HCT116 cells were used to create a xenograft flank tumor model system in immunodeficient mice. Xenograft models in athymic mice were selected to enable direct evaluation of the human cancer cell-specific therapeutic response while minimizing immune-related variability. This controlled setting allows a clearer mechanistic assessment of nanoparticle pharmacokinetics and radiosensitization prior to expansion into immunocompetent syngeneic tumor models. Upon observation of visible tumor growth, a single intratumoral injection was administered 24 h prior to selective IR. Selectivity of the radiation field was achieved using a lead shield that only exposed the right flank of the animal where the tumor was located. The effects of the treatment on tumor growth and volume were monitored for 12-days with four mice per group (Figure 6A–E). The combination system (NP+pM) not only inhibited tumor growth but also led to tumor volume regression over a period of 12 days with a relatively low radiation dose of 4 Gy in a single fraction. A comparison between the groups’ mean relative tumor volume showed that treatment with NP+pM with and without radiation significantly outperformed radiation alone (adjusted p-value of 0.0004 through one-way analysis of variance and Tukey’s multiple comparison test).

**Figure 6:**
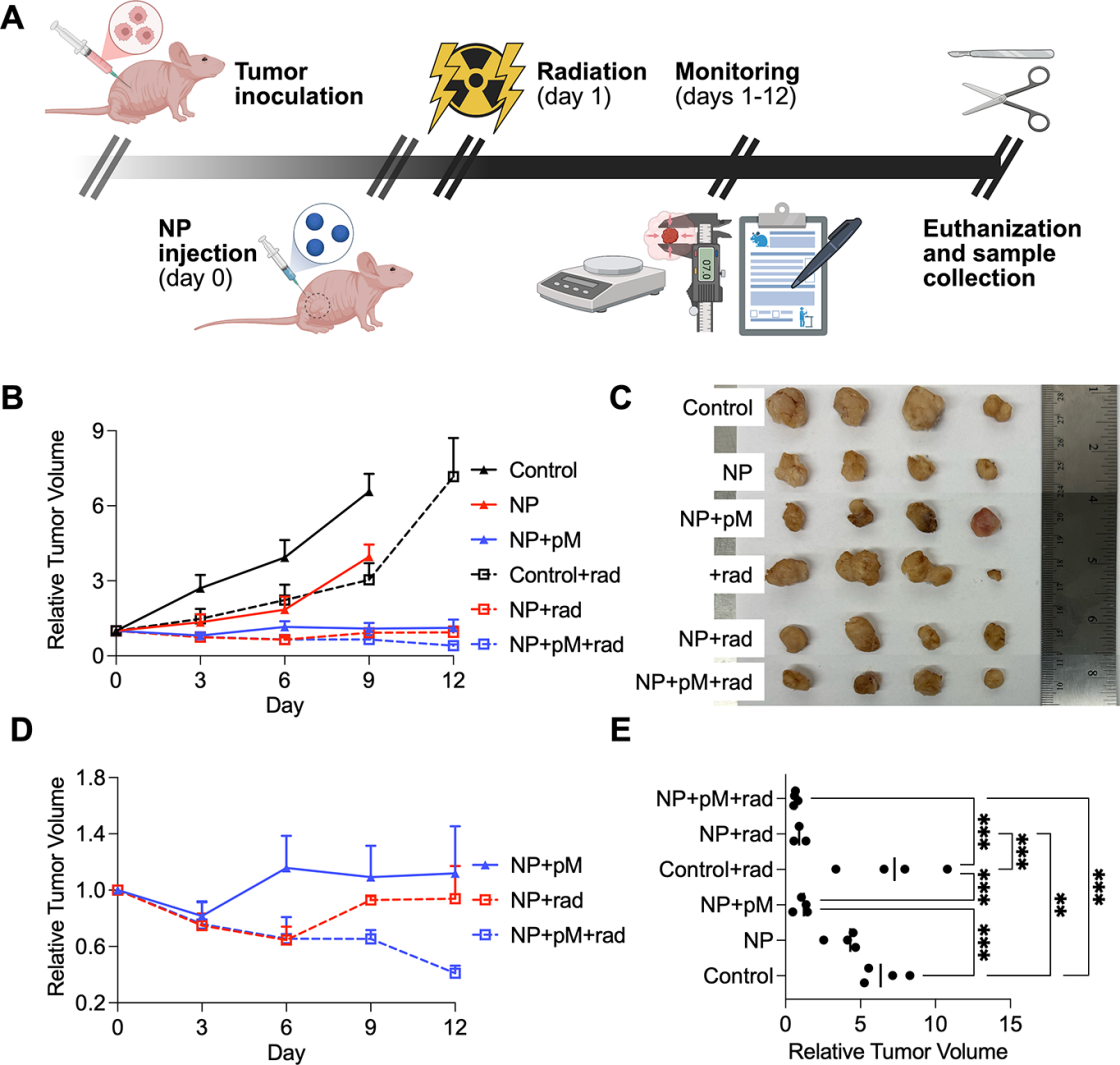
In vivo tumor xenograft model study results. (A) Procedure timeline. (B) Mean group relative tumor volume over duration of treatment period. (C) Excised tumors post study. (D) Mean group relative tumor volume of groups that exhibited tumor regression during treatment period. (E) Relative tumor volume of groups at the study end point with statistical analysis. Error bars depicted as standard error of the mean (*n* = 4).

The effects of NP+pM combined with radiation showed no evidence of toxicity-related safety concerns. None of the animals exhibited a decrease in the mean body weight, supporting the biocompatibility of the treatment (Figure 7A). Complement activation in serum samples was examined using a C5a enzyme-linked immunosorbent assay as a measure of innate immune and inflammatory responses (Figure 7B). C5a production did not significantly differ between the treatment and control groups, demonstrating that the combination system did not induce a detectable change in innate immunogenic response. General tolerance was supported by the absence of adverse effects in healthy mice treated with NP+pM, regardless of radiation exposure. Follow-up hematology and serum chemistry panels of blood samples, collected in replicate per group (Figure 7C–E) corroborated these observations. Creatinine and blood urea nitrogen, which are markers of renal function, remained within normal physiological ranges, indicating preserved kidney function. Liver health was similarly unaffected, as shown by stable alanine aminotransferase and aspartate aminotransferase levels relative to the baseline. Complete blood count with differential analysis further confirmed that the treatment did not disrupt hematological balance.

**Figure 7:**
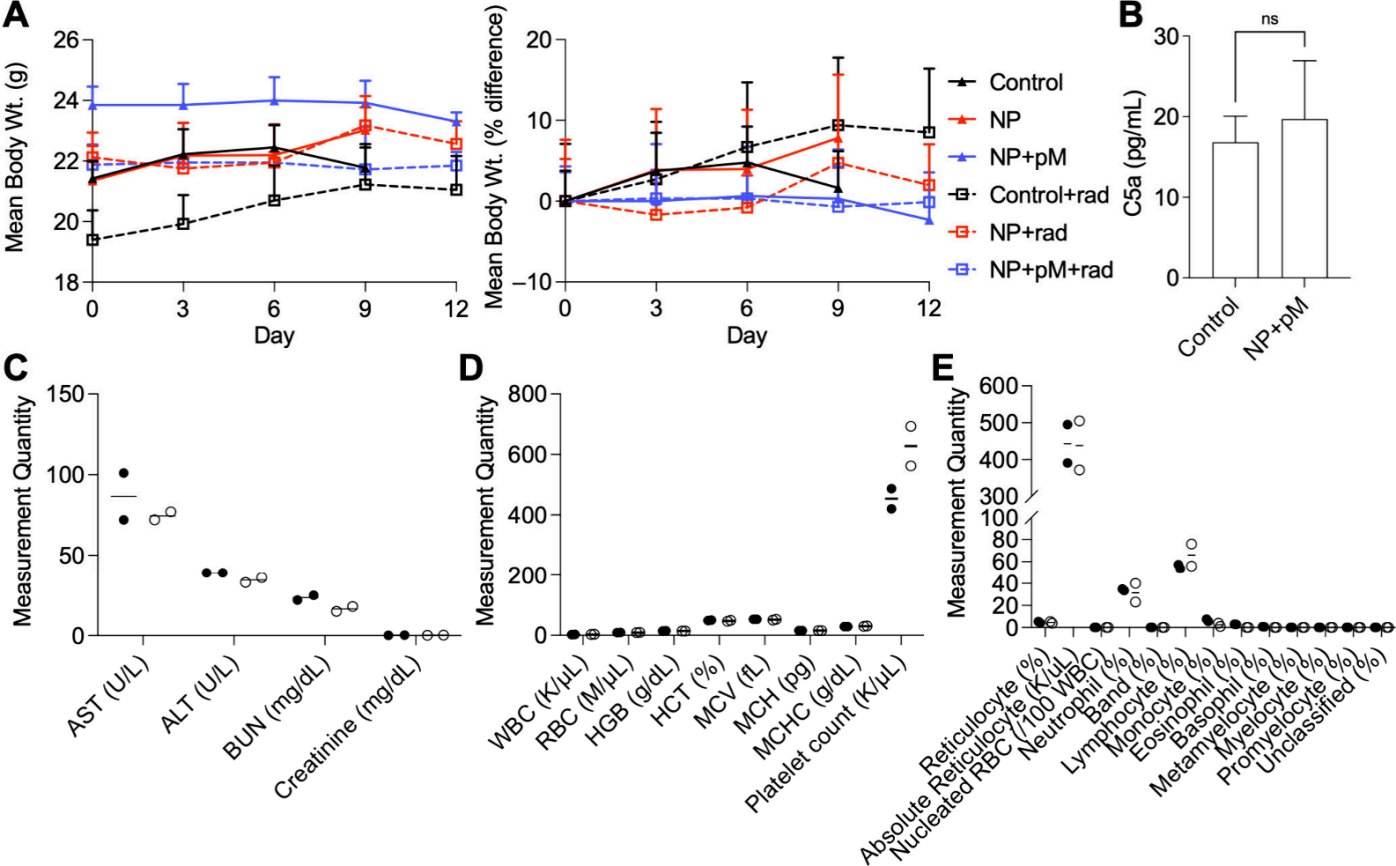
In vivo model biocompatibility, immunogenic, and hemocompatibility studies. (A) Treatment effect on body weight (*n* = 4). (B) Complement C5a enzyme-linked immunosorbent assay (*n* = 3). (C) Clinical blood parameters. (D) Differential blood test. (E) Complete blood count.

The presence of HfNPs in the tissue sections was further confirmed by hyperspectral imaging, in which a spectral fingerprint was obtained for individual pixels^32^ (Figure 8A, B). The spectral profile of the HfNPs was depicted from the particles and is attributed to the HfNPs being tethered or dislodged from the HfLNPs, which corresponds to absorption in the red region. This analysis is complementary to other in vivo biodistribution studies because it provides insight regarding spatial distribution within tissue samples. The importance of assessing the spatial behavior in the in vivo model (i.e., homogeneous versus heterogeneous distribution) is critical to the physical mechanism of dose enhancement underlying the high-Z radiosensitization as the biological effects are more widespread across the tumor.

**Figure 8:**
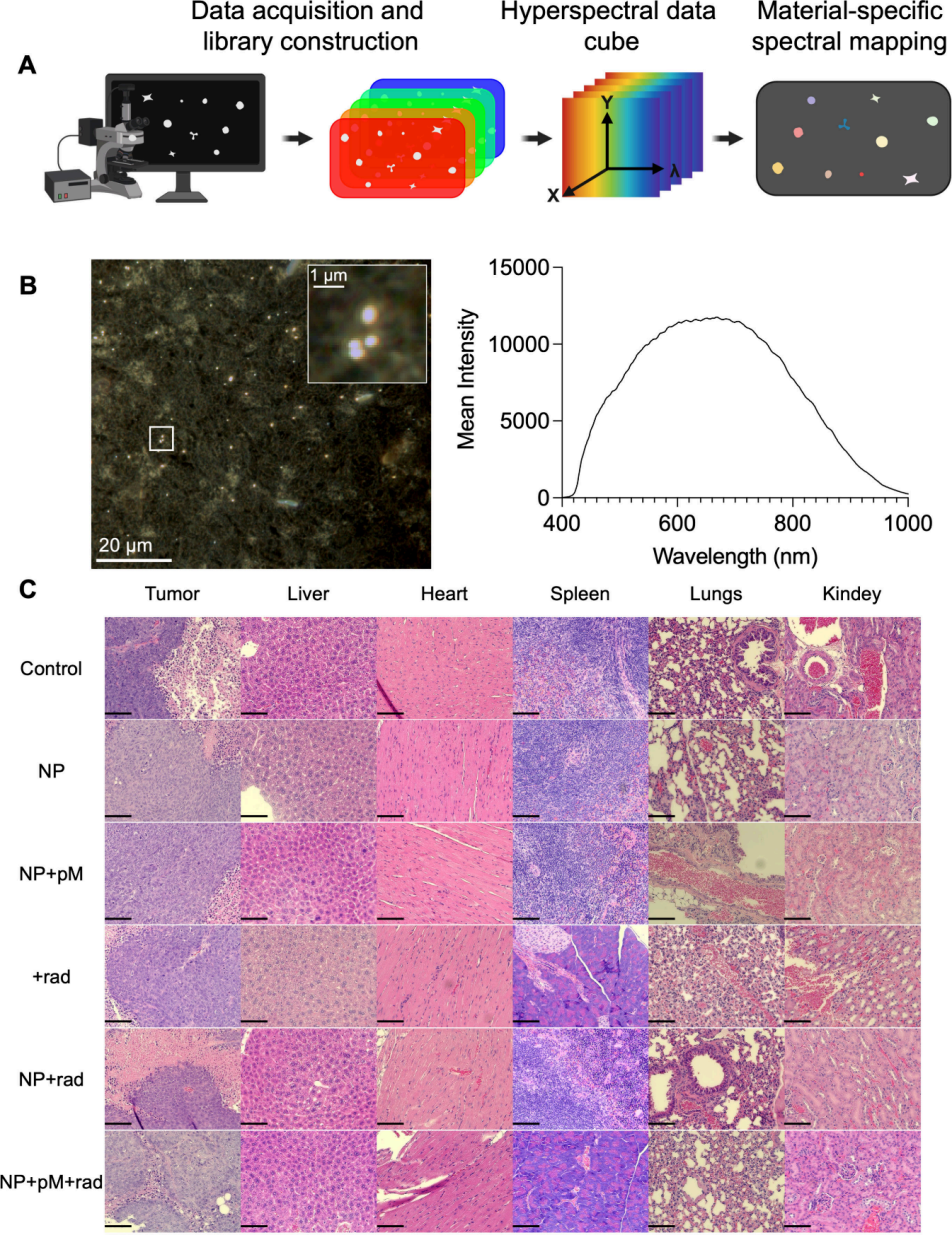
Tissue-level analyses. (A) Hyperspectral imaging principle and workflow diagram. (B) Hyperspectral imaging of particles in the tissue section. Bright spots correspond to particle scattering. Right plot provides mean spectral information for particles included in the magnified inset image. (C) Hematoxylin and eosin–stained histology slides. Inset scale bar = 100 um.

A tissue-level histological examination of the major organs (heart, lung, liver, spleen, and kidney) and tumor tissues was performed to assess the systemic biocompatibility and therapeutic efficacy following treatment administration (Figure 8C). No structural abnormalities or treatment-associated damage was observed, reinforcing that NP-pM is systemically well-tolerated and remains biocompatible under radiation-based therapeutic conditions. Hematoxylin and eosin-stained slices of nontumor tissues displayed normal histological architecture in all treatment groups. The cardiac tissue exhibited intact muscle fibers devoid of inflammatory infiltration or necrosis. The pulmonary sections exhibited an intact alveolar architecture and were devoid of edema or fibrosis. The hepatic tissue exhibited a distinct lobular architecture with prominent central veins and sinusoids and displayed no signs of hepatocellular enlargement or vacuolation. The renal cortex and medulla exhibited typical glomerular and tubular architectures without detectable degeneration or necrosis, whereas the splenic tissue demonstrated an intact organization of white and red pulp. These data collectively indicated a lack of acute or chronic systemic toxicity and aligned with the blood biochemistry results, demonstrating normal hepatic and renal functions. Tumor tissues from the NP-pM–treated and NP-irradiated groups displayed significant morphological alterations indicative of radiation-induced cytotoxicity. The tumor cells exhibited nuclear condensation, karyorrhexis, and extensive cytoplasmic vacuolation, indicating cell death and disintegration. Compared with the control and radiation-only groups, combination treatment led to significant tumor necrosis and decreased cellular density, indicating increased DNA damage and diminished repair capability due to the synergistic effects of high-Z radiosensitization and mirin-mediated HR inhibition. Histopathological findings indicate that the NP-pM nanosystem selectively triggers tumor-specific cell death while preserving normal tissues, thus affirming its potential as a safe and effective platform for targeted chemoradiotherapy.

Spectral photon-counting computed tomography imaging (Figure 9A–C), capable of material-specific attenuation identification,^33–37^ and ICP-MS of dissected organs (Figure 9D) was used to assess biodistribution 12 days post injection. Imaging and experimental quantification revealed a distribution pattern dominated by sustained tumor retention.

**Figure 9:**
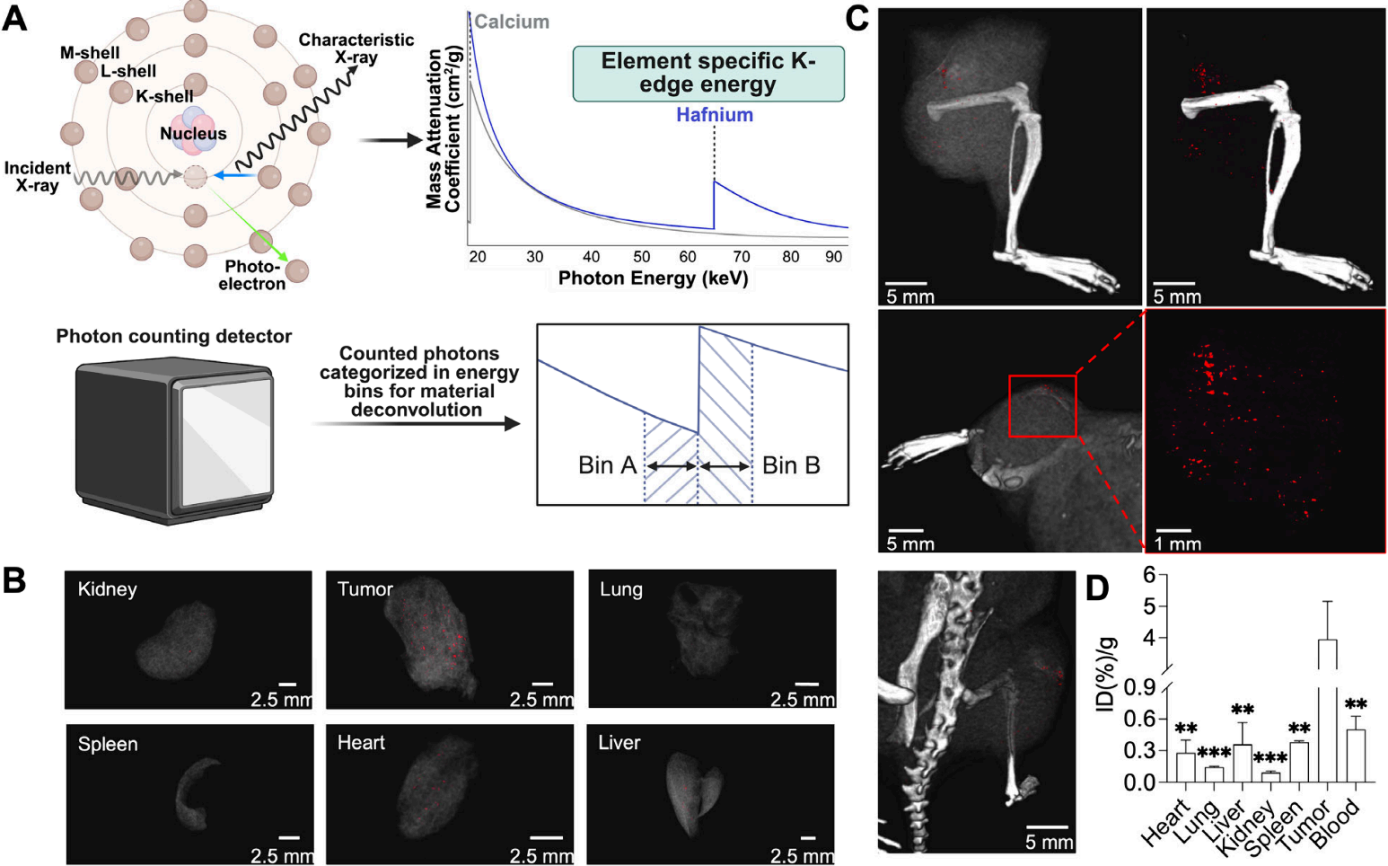
Biodistribution assessed through SPCCT (A–C) and ICP-MS (D) after 12 days. (A) Working principle of SPCCT enabling material-dependent CT signal decomposition. (B) Representative images of excised organs. (C) Representative image of mouse with injected right flank tumor. Red signal intensity corresponds to the presence of Hafnium. (D) Injected dose percent per gram of HfNPs 12 days after injection (*n* = 3).

ICP-MS showed that most of the measurable hafnium still resided within the tumor mass, far exceeding the levels detected in other tissues (Figure 9D). The liver and heart exhibited the highest concentrations of the administered dose on a per-gram basis, excluding the tumor. This organ profile corresponds to hepatic processing, which is the principal route of elimination. SPCCT imaging was predominantly aligned with the ICP-MS findings, whereas it exhibited a notable hafnium signal in the heart, which corresponded to increased levels identified in the blood. Additionally, SPCCT imaging demonstrated a significant Hf signal within the tumor, suggesting the prolonged retention of nanoparticles in the tumor microenvironment. The extended intratumoral retention of the nanoparticles was ascribed to the continued distribution of HfNPs from the injected HfLNPs. The NPs tethered in the lipid bilayer exhibited a decreased diffusion-mediated clearance and facilitated prolonged retention at the injection site. HfLNPs facilitated protracted radiosensitization, as high-Z NPs remain in the tumor tissue for a lengthy period, thereby enhancing local radiation dose deposition and ROS formation across an extended treatment window.

## Discussion

Although inorganic nanoparticles offer several advantageous physicochemical properties, these materials experience toxicity issues, particularly dissociation into reactive cations. In contrast, soft-material-based nanoparticles, such as liposomes and LNPs, are inherently safer because of their biomimetic nature but lack the capability for high-Z radiosensitization. In this study, we sought to leverage the benefits of both materials to minimize nonspecific toxicity without compromising their radiosensitizing ability.

The combined NP–prodrug system produced a strong radiation-activated response in both in vitro and in vivo assays. The nontoxic behavior of NPs without IR is due to their physicochemical stability and extremely crystalline structure, which prevents the release of damaging metallic ions, which are often the primary source of inherent cytotoxicity in inorganic NPs. This demonstration of the biocompatible nature coupled with the uncompromised cytotoxicity of the prodrug compared with the standard form supports the practicality of the dual-pronged strategy. Moreover, a larger particle capsule size prevents the rapid renal clearance of ultrasmall radiosensitizing particles, promoting long-term therapeutic effects. A prior review by Jain et al. suggested that nanoparticles in the 30–200 nm range accumulated most effectively in solid tumors.^38^ Experimental data also showed that PEGylated nanoparticles of approximately 100 nm were physically trapped within the extracellular matrix, whereas smaller particles (∼20 nm) escaped more easily.^39,40^ Therefore, we attribute the prolonged retention time of the Hafnia particles to their transport via a larger LNP. This may maintain a consistent outflow from the tumor, enhance the predictability of safety, and sustain therapeutic effects.

NBTXR3 is the first-in-class high-Z radiosensitizer based on advancement to clinical trials. Thus, a brief comparison between this and the nanoscale platform at a similar stage in their respective timelines is warranted. The first publication documenting NBTXR3 utilized the same cell line tumor xenograft model and saw a similar trend in diminishing tumor volumes across a comparable time scale, albeit with double the total radiation dosage (8 Gy).^41^ Although not a direct head-to-head experimental comparison, this lends credibility to the coupled-prodrug strategy, allowing for lower radiation doses without compromising efficacy.

This study serves as a preliminary demonstration of the integrated prodrug–radiosensitizer method; hence, comprehensive assessments of the systemic distribution, pharmacokinetics, and temporal dynamics will be essential in the future. Our findings demonstrate that the HfLNP platform is physically stable, biologically compatible, and proficient in providing dual functionality: high Z-mediated radiosensitization and effective prodrug activation in tumor-relevant settings. The nanosystem demonstrated significant tumor growth inhibition in vivo by enhancing the radiation dosage and inhibiting DNA repair mechanisms, thereby confirming its therapeutic efficacy.

## Conclusion

The design and development of the prodrug-bearing and ultrasmall Hafnium oxide-decorated LNPs were confirmed through comprehensive characterization. Prodrug activation has been evaluated mechanistically, confirming specific activation in response to the presence of enzymes and decreased pH. Moreover, the biological function of the prodrug has been verified in an in vitro model, and a comparison with the active drug molecule has identified the functionality to be unaltered. The proof-of-concept for the dual approach and synergistic effects, namely, Hafnia radiosensitization-mediated DSB induction and DSB repair inhibition, has been demonstrated in both in vitro and in vivo models. The nanosystem showed the capacity to sustain therapeutic efficacy at low radiation doses and displayed markedly enhanced tumor-reduction effects compared to radiation alone in vivo. Furthermore, in vivo follow-up studies assessing the immunogenic response and biocompatibility corroborate the safety of this therapeutic augmentation technique.

## Methods

### HfNP Synthesis

Nanoparticles were synthesized using a modified precipitation method based on established procedures found in literature.^42–44^ HfCl_4_ is dissolved in deionized water (0.1 M), and a concentrated NaOH solution (3 equiv) is added dropwise under vigorous stirring to form a white hafnium hydroxide suspension, which is then aged at elevated temperature (80 °C) to yield HfO_2_ nanocrystals. The solid is collected by centrifugation, washed repeatedly with water and ethanol to remove salts and unreacted precursors, and then gently dried to a damp cake while avoiding complete dehydration to limit irreversible aggregation. The wet HfNP cake is immediately dispersed into a minimal volume of ethanol by sonication, producing a concentrated HfNP dispersion that can be directly introduced into the lipid formulation step.

### HfLNP Preparation

For HfLNPs, a lipid mixture consisting of 5 mol % prodrug, 12.5 mol % cholesterol, 82 mol % L-α-phosphatidylcholine, and 0.5 mol % 18:0 PEG2000-PE is dissolved in chloroform:ethanol mixture in a round-bottom flask, with total organic lipid loading set to 8 μmol per mg of HfNPs. The ethanol dispersion of damp HfNPs is added directly to this lipid solution under sonication to distribute the inorganic phase within the organic matrix, and the mixed solution is then evaporated under a gentle stream of inert gas to form a uniform composite thin film, followed by vacuum drying to remove the residual solvent. Hydration is performed with 1X PBS buffer while vortexing and sonicating to drive self-assembly of lipid bilayers around and with the HfNPs, yielding HfLNP dispersions, and multiple freeze–thaw sonication-vortex cycles were performed. Denser, Hf-loaded vesicles are centrifuged, washing, and resuspension, producing HfLNPs with HfNPs exhibiting embedded within or closely associated with the lipid bilayer, PEGylated outer surfaces, and coloaded prodrug, with colloidal stability.

### EM Characterization

TEM characterization procedure followed standard protocols.^45,46^ 3.5 μL of NP dispersion (3.5 μL) was drop cast onto carbon-coated copper grids and blotted to dry. Imaging, SAED, and EDS was performed on a Talos F200X (Thermo Fisher Scientific) TEM operated at 200 kV and equipped with a Ceta CMOS CCD camera and four in-column SDD Super-X detectors. For EDS, elemental maps were acquired for approximately 5 min (beam current = 0.12 nA) and analyzed using Velox software. ImageJ was used for an additional image analysis. Osmium tetroxide 2% aqueous solution was used for positive staining to visualize the liposomal assembly.

### XRD

Powder X-ray diffraction was conducted on an Empyrean III diffractometer (Malvern PANalytical Inc.) equipped with a Cu Kα source. Diffractograms were collected from 5° to 70° in 2θ with a step size of 0.026°.

### DLS and Zeta Potential

Dynamic light scattering and zeta potential analyses were carried out on a Malvern Zetasizer Nano series instrument (633 nm laser). Samples were loaded into disposable cuvettes. For DLS, three measurements per sample were acquired and averaged using Zetasizer software. Zeta potential measurements were performed by using folded capillary zeta cells on the same instrument.

### Prodrug Synthesis

The esterification between Mirin and PAz-PC proceeded via a modified EDC coupling reaction. The carboxylic acid on PAz-PC was activated with EDC and DMAP, followed by nucleophilic attack by the deprotonated Mirin phenol; NHS was used to facilitate phenol deprotonation. DMSO served as the reaction solvent. Reaction progress was initially monitored by thin-layer chromatography (TLC) using a mobile phase of 6% (v/v) methanol in chloroform. The crude product was purified by liquid–liquid extraction in a separatory funnel with chloroform as the organic phase. Successful conjugation and purification were confirmed by ^1^H NMR and ESI-MS.

### Prodrug Loading

ProMirin loading into the NP coatings was quantified using MS/MS UHPLC, with loading efficiency (%) reported as a function of nanomaterial morphology. Supernatants from the lipid washing steps were diluted to 90% methanol to disrupt the lipid assemblies before MS analysis.

### Irradiation Settings

All irradiation (in vitro and in vivo) was carried out using an X-Rad320 instrument (320 kV, 12.5 mA). Doses were delivered as a single fraction at a rate of 87 cGy/min. The source-to-surface distance was 50 cm, and the field size was approximately 20 × 20 cm. Previous dosimetry studies investigating the field homogeneity with the specific X-ray model and configuration support a homogeneous dose distribution with a 4.4% normalized signal standard deviation for total doses within the range used in this study.^47^ After irradiation, cell culture plates were returned to the incubator, and animals were placed back into an autoclaved housing. For in vivo tumor treatments, animals were immobilized in a holding fixture behind a lead shield with an aperture that selectively exposed the right flank tumor.

### In Vitro Studies

HCT116 colorectal carcinoma cells (ATCC) were maintained in McCoy’s 5A medium at 37 °C and 5% CO_2_ and passaged roughly every 48 h.

### MTT Assay

Baseline cytotoxicity was evaluated using an MTT assay.^48–51^ Cells were seeded at 1 × 10^4^ cells per well in 96-well plates and allowed to adhere overnight. Groups were assayed in n = 4. Media were replaced, and treatment solutions or PBS (1×) vehicle were added at the appropriate dilutions, followed by gentle agitation to ensure mixing. Plates were then incubated for 3 days. Subsequently, MTT solution was added to yield a final concentration of 5 mg/mL in each well. Plates were shaken at 900 rpm for 5 min and incubated for 4 h. Media were then removed, and formazan crystals were solubilized in 150 μL of DMSO per well. Absorbance at 540 nm was measured on a plate reader. All groups were run in triplicate. Cell viability percent was calculated as below in eq 1.

1 $$\left[\frac{{Abs}_{\mathrm{treatment}}-{Abs}_{\mathrm{blank}}}{{Abs}_{\mathrm{mean control}}-{Abs}_{\mathrm{blank}}}\right]\times 100$$

### Clonogenic Assay

Clonogenic survival was measured following Franken et al.^52^ An initial assay with no treatment was used to determine the optimal cell seeding density. For subsequent experiments, HCT116 cells were seeded in six-well plates at 200–400 cells/well for negative controls and allowed to adhere overnight; treated groups were seeded at densities adjusted for expected toxicity. After attachment, treatment solutions were added, and plates were returned to the incubator for predetermined internalization times prior to irradiation. After radiation, cells were incubated for an additional 7 days (8 days total from seeding), then washed with PBS 1×, fixed, and stained with 2 mL/well of 6.0% (v/v) glutaraldehyde and 0.5% (v/v) crystal violet for 30 min. In all assays, groups were prepared in n = 4 ± 2.

### ICP-MS Uptake Kinetics

To examine NPs internalization, HCT116 cells were seeded in duplicate 6-well plates at 1.5 × 10^5^ cells/well and incubated overnight. Treatment solutions were added and incubated for 2, 4, or 6 h. At each time point, media were removed, and wells were washed three times with PBS 1× to remove extracellular particles. Cells were detached with 1 mL/well Trypsin-EDTA (0.25%) for 3 min, pelleted, resuspended in 0.5 mL of Milli-Q water, and counted. Cell suspensions were transferred to glass vials for acid digestion using 0.5 mL ICP-grade HNO_3_ and left loosely capped in a fume hood for 72 h. Samples were then diluted to 10 mL with Milli-Q water and analyzed by ICP-MS. Stock treatment solutions were digested in parallel. Hafnium content was normalized to the cell number. Groups were assayed in n = 4 ± 2.

### RAD51 Prodrug Mechanism Assay

The prodrug’s mechanism of action was evaluated using a RAD51 foci assay with confocal imaging. Approximately 3 × 10^5^ cells were seeded and allowed to adhere overnight. The following day, drug, prodrug, or vehicle was added, and the mixture was incubated for 4 h before irradiation (4 Gy). Cells were then incubated for an additional 6 h. Cells were washed with PBS 1× and fixed in 4% paraformaldehyde in PBS 1× for 15 min, rinsed with TBS, and permeabilized with 0.25% Triton X in TBS for 10 min. Blocking was performed overnight at 4 °C in 1× Blocker in TBS with gentle agitation. The next day, cells were incubated with primary RAD51 antibody (1:1000 in 1× Blocker/TBS) for 2 h at room temperature, washed three times with 0.05% Tween-20 in TBS (10 min each), and then incubated with FITC-conjugated secondary antibody (1:1000 in 1× Blocker/TBS) for 1 h. After three additional washes with 0.05% Tween-20 in TBS and a final TBS rinse, the cells were mounted with DAPI-containing media and imaged on a Zeiss LSM 880 confocal microscope. RAD51 foci were quantified by using the CellProfiler “Speckle Counting” pipeline.

### ROS and Protein Normalization Assays

Relative ROS levels were measured using a DCFDA assay and normalized to protein content via a TCA-SRB assay.^53^ Cells were seeded at 20,000–30,000 cells/cm^2^ in black 96-well plates (≈6400 cells/well for a 0.32 cm^2^ well area) and allowed to adhere overnight. Media were replaced, and NPs treatments were added. Plates were incubated overnight before irradiation. Following radiation, media were replaced with DCFDA working solution and incubated for 30 min at 37 °C. Wells were washed once with PBS 1×, and PBS 1× supplemented with 10% FBS and 1% PS was added. Fluorescence was immediately measured (Ex 494 nm and Em 522 nm). The TCA-SRB assay (Ex, 565 nm; Em, 586 nm) was then performed on the same wells. Cell-free background signals were subtracted, and both DCF and SRB intensities were expressed as percentages of untreated controls; DCF:SRB ratios were then calculated. Groups were assayed in n = 3.

### γ-H2AX Flow Cytometry

Radiation-induced DNA double-strand breaks were quantified by γ-H2AX flow cytometry. HCT116 cells were seeded at 1 × 10^6^ cells/well in 6-well plates, allowed to adhere overnight, and then treated with either 50 μg/mL hafnium NPs or 3 μM proMirin. After 2 h, cells were irradiated with 4 Gy and incubated for an additional 1 additional hour. Cells were washed with PBS 1×, detached with 1 mL/well Trypsin-EDTA (5×), pelleted, washed again in PBS 1×, and fixed in ice-cold 70% ethanol. Fixed samples were stored at – 20 °C for less than 2 weeks, and all samples were processed on the same analysis day. For staining, 1 mL of Tris-buffered saline (TBS, pH 7.4) was added to each sample, and cells were pelleted and resuspended in 1 mL of TBS containing 4% FBS and 0.1% Triton X-100. After a 10 min rehydration, cells were centrifuged and resuspended in 0.2 mL of mouse monoclonal phospho-histone γH2A.X antibody (1:500 in TST) and agitated at 225 rpm for 2 h. Samples were washed, then resuspended in 200 μL Antimouse IgG (H+L), F­(ab’)_2_ (Alexa Fluor 488, 1:200 in TST), and shaken for 1 h. Following another wash, cells were resuspended in TBS containing DAPI. Flow cytometry was performed by using a Coulter Elite cell sorter. Groups were assayed in n = 3.

### In Vivo Model

All animal work conformed to protocols approved by the Pennsylvania State University Institutional Animal Care and Use Committee. Immunodeficient mice (JAX stock no. 002019) were obtained at 3 weeks of age, acclimated for 1 week, and maintained in autoclaved cages with sterile food and water. Groups for tumor volume and animal weight were assayed in n = 4.

Tumors were induced by subcutaneous injection of 3 × 10^6^ HCT116 cells in a 1:1 mixture of culture medium and Matrigel into the right flank. Cell cultures were screened for murine pathogens before injection. Treatments were administered approximately 10 days after tumor induction, once palpable superficial tumors formed. Each tumor received a single intratumoral injection of the assigned treatment. Radiation, where applicable, was delivered 24 h after injection. Animals were monitored throughout the study and euthanized at predetermined end points. At euthanasia, tumor and major organs (heart, lungs, spleen, kidney, liver) as well as urine and feces were collected and stored in 4% paraformaldehyde at 4 °C. Tissue preparation for ICP-MS followed modified standard protocols.^54^ Samples were rinsed in PBS 1×, blotted dry, weighed, and digested in 70% HNO_3_ for 24 h until they were fully liquified. Digests were then heated to dryness and reconstituted in 10 mL of 2% HNO_3_. Solutions were filtered prior to ICP-MS; if volume was lost during filtration, additional 2% HNO_3_ was added to restore the final volume to 10 mL. Groups were assayed in n = 3 for ID%/g. For SPCCT, euthanized animals were frozen and stored at – 80 °C for ≤ 3 days before imaging. Excised organs were stored in 4% paraformaldehyde, embedded by dipping in paraffin wax, sectioned using a microtome, and mounted on glass slides. Paraffin was removed, and sections were rehydrated and stained using a standard hematoxylin and eosin protocol. Complement C5a ELISA assay was performed using a kit following vendor’s provided protocol (Cat. No.: EMHC) and groups were evaluated in n = 3. Hematology and serum chemistry assays were assessed in n = 4 ± 2.

### Data Analysis and Plotting

Data visualization and statistical tests were performed in GraphPad Prism 10.2.0.335 for macOS. Unless noted otherwise, comparisons were made using either Student’s *t* test or one-way ANOVA. Significance levels were defined as ns, *P* > 0.05; *P ≤ 0.05; **P ≤ 0.01; ***P ≤ 0.001; ****P ≤ 0.0001. Error bars depict SD unless otherwise noted as the SEM.

## Supplementary Material



## Notes

Additional experimental details and supplementary results referenced in the paper, including zeta-potential, DLS, TEM, ESI-MS, ^1^H NMR, UHPLC-MS/MS calibration curves, and stimuli-dependent prodrug activation (PDF)
