# Supplementary material for: A Dual-Pronged Hafnia–Prodrug–Lipid Nanoplatform Coupling Radiosensitization and DNA Homologous Recombination Inhibition
Source: ACS Nano Med. 2026 Apr 14;1(6):1456–68. doi: 10.1021/acsnanomed.6c00022 (PMC13352939; doi:10.1021/acsnanomed.6c00022)
Supplement: Supplementary file 1 [file nm6c00022_si_001.pdf]

Supporting Information

A Dual-Pronged Hafnia-Prodrug-Lipid Nanoplatform Coupling Radiosensitization and DNA Homologous Recombination Inhibition

David Skrodzki[a,†], Pranay Saha[b,†], Matthew Molinaro[c], Nada Maher[b], Shraddha Krishnakumar[d], Nivetha Gunaseelan[d], Mecit Altan Alioglu[b], Oguzhan Colak[b], Gabriela Cepeda[d], Parikshit Moitra[b], Timothy M. Fan[g,h], Dipanjan Pan*[,a,b,c,d,e,f]

[a] Department of Materials Science and Engineering, The Pennsylvania State University, University Park, PA 16802, USA

[b] Department of Nuclear Engineering, The Pennsylvania State University, University Park, PA 16802, USA

[c] Department of Engineering Science and Mechanics, The Pennsylvania State University, University Park, PA 16802, USA

[d] Department of Biomedical Engineering, The Pennsylvania State University, University Park, PA 16802, USA

[e] Huck Institutes of Life Sciences, 101 Huck Life Sciences Building, University Park, PA 16802, USA

[f] Department of Chemistry, The Pennsylvania State University, University Park, PA 16802, USA

[g] Department of Veterinary Clinical Medicine, University of Illinois Urbana-Champaign, Urbana, IL, 61802, USA

[h] Cancer Center at Illinois, University of Illinois Urbana-Champaign, Urbana, IL, 61802, USA

[†]Equal contribution

*Corresponding author: Dipanjan Pan, Email: dipanjan@psu.edu

Table of Contents

Contents (Fig. No.)..**Pg. No.**

Bare particle stability in water (S1)..S3

DLS by intensity of HfLNP (S2)...S3

Positive-stained TEM image of HfLNP (S3)...S4

ESI-MS of prodrug synthesis product (S4)...S5

Proton NMR of prodrug synthesis product (S5)...S6

UHPLC-MS/MS prodrug calibration curves (S6)..S6

Stimuli-induced prodrug activation study (S7)...S7

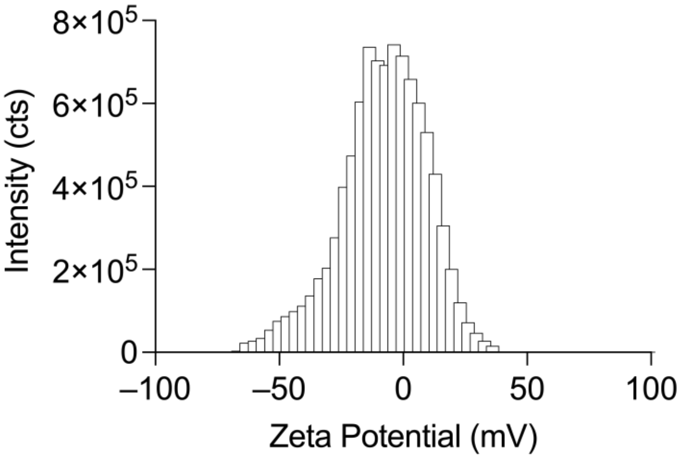

Figure S1. Zeta-potential of as synthesized NPs re-suspended in water demonstrating hydrophobic-like nature.

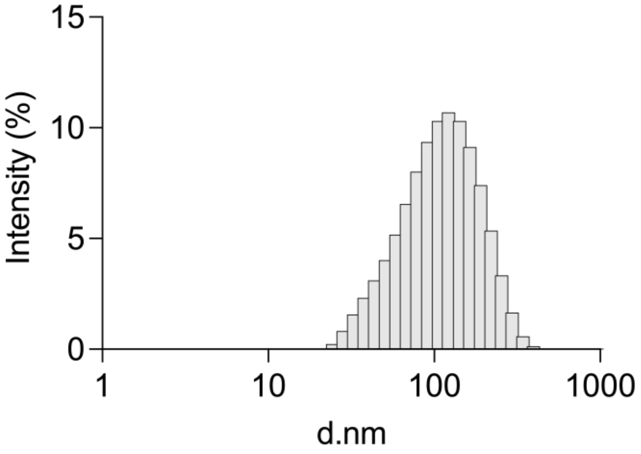

Figure S2. Hydrodynamic size of HfLNP by intensity.

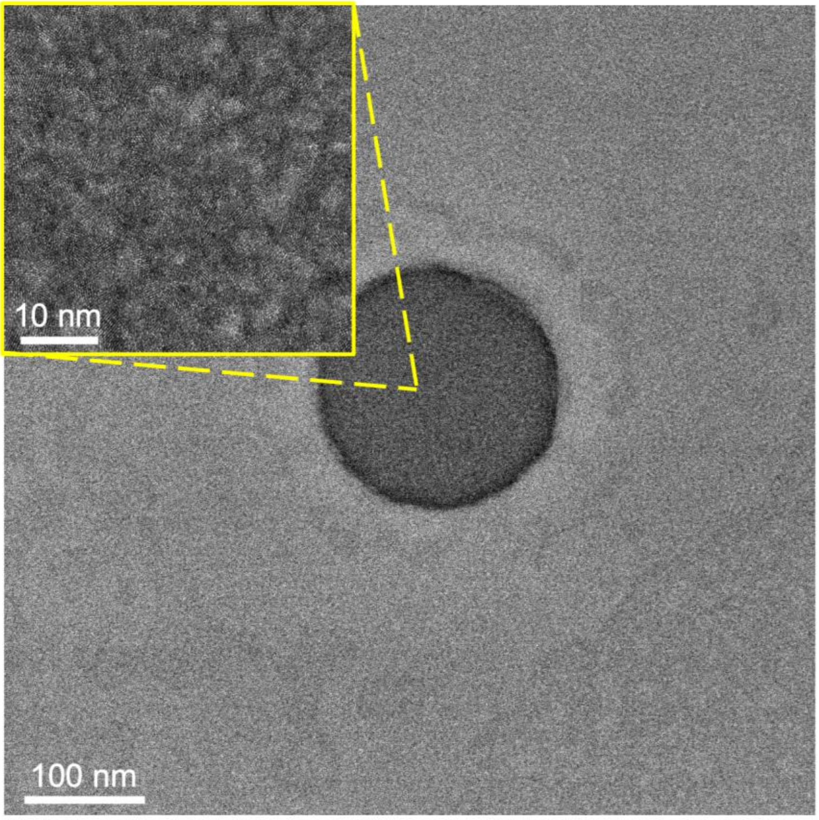

Figure S3. Positive-stained TEM image of HfLNP with inlaid HRTEM depicting lattice fringes with various orientations corresponding to tethered HfNPs.

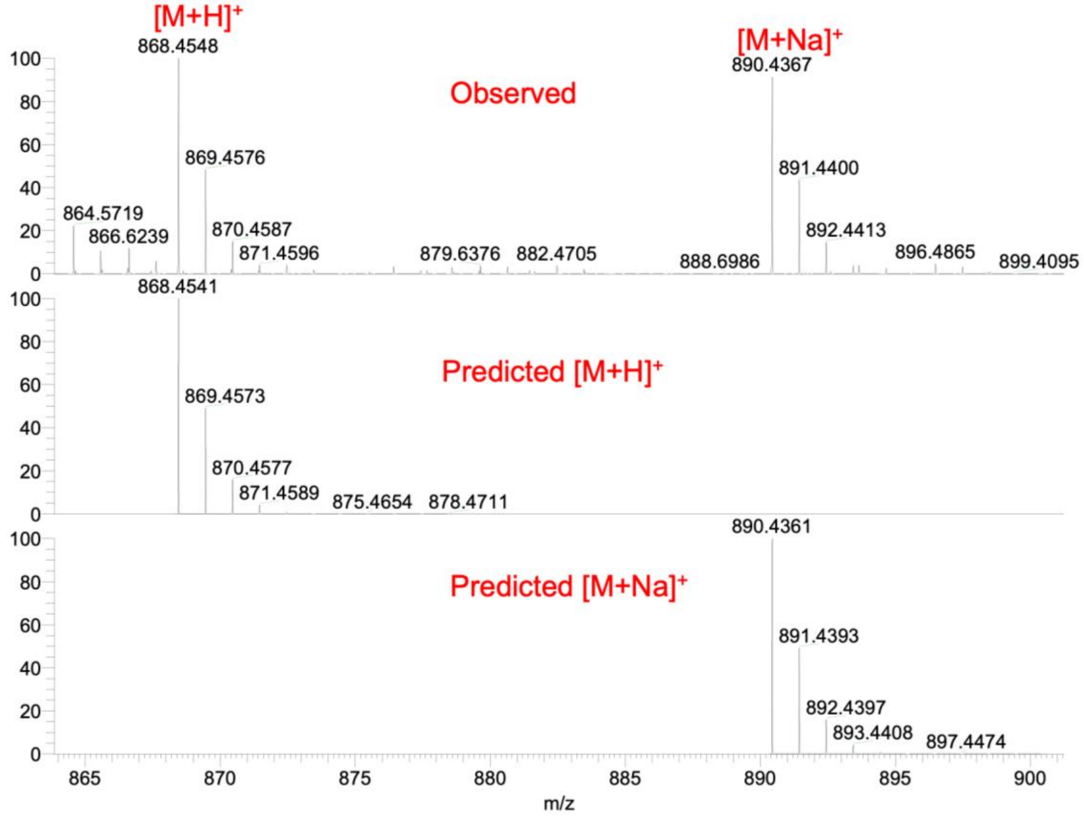

Figure S4. ESI-MS of prodrug synthesis product compared to the predicted m/z ratio of hydrogen and sodium ion-adducts.

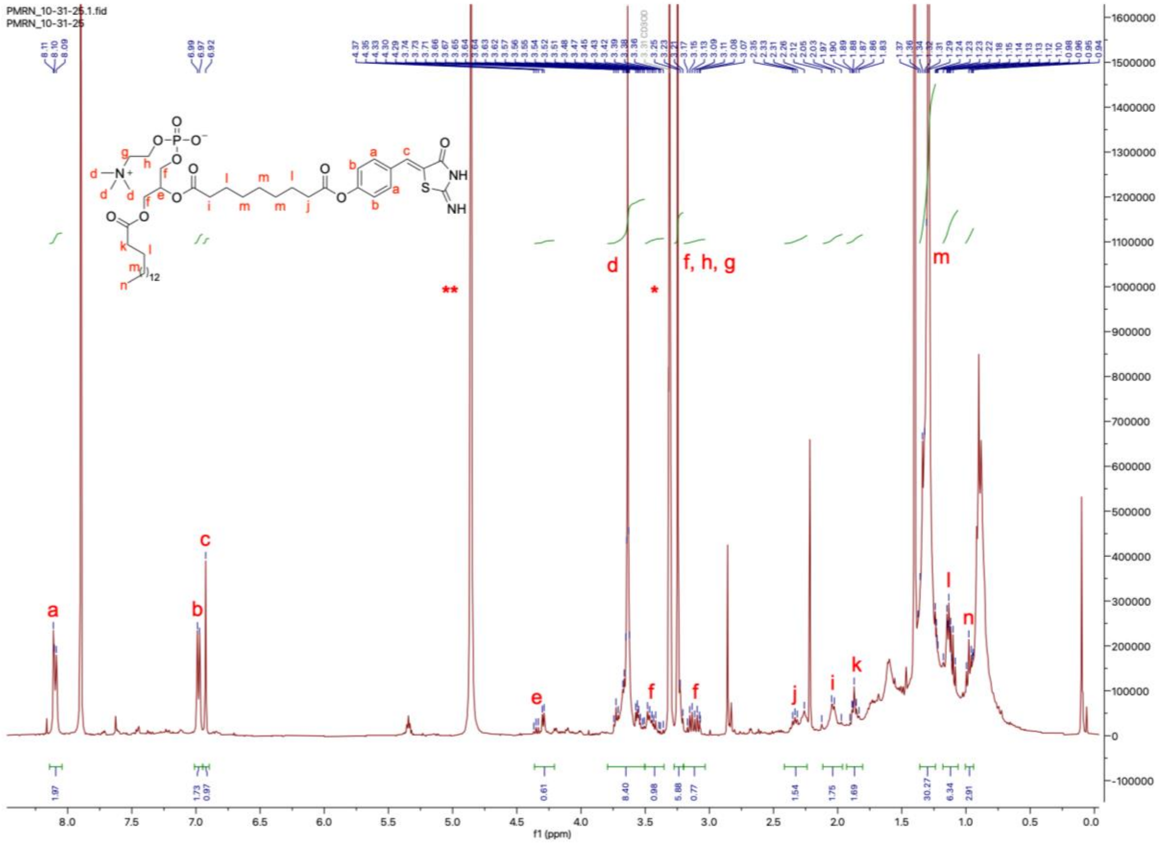

Figure S5. Prodrug synthesis product ^{1}H NMR in methanol-d4. Solvent signal (*) and solvent water trace impurity (**) indicated.

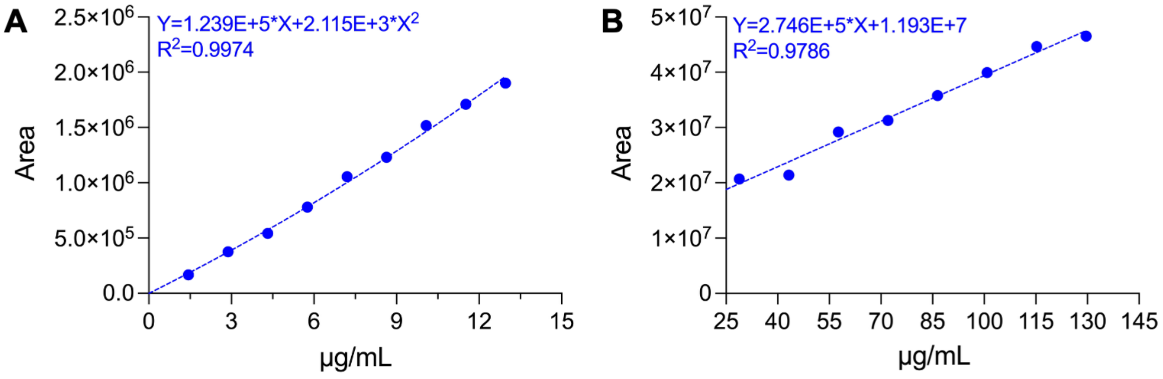

Figure S6. Prodrug calibration curves used during UHPLC-MS/MS to quantify particle drug loading. (A) Nonlinear fit for 1.5-15 μg/mL. (B) Linear fit for 15-100 μg/mL.

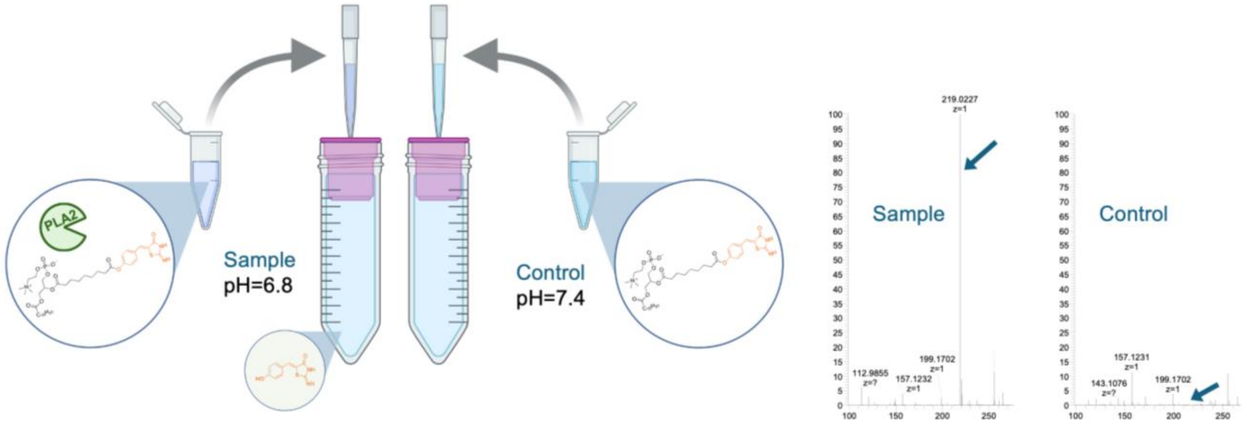

Figure S7. Prodrug activation study results depicting ESI-MS of dialysate from control (right) (pH=7.4) incubated without enzyme and sample (left) (pH=6.8) incubated with phospholipase A2 (15 IU/mL). The Mirin peak is only observed in the presence of enzyme at lowered pH.
